# Mechanistic insight into the anti-alternaria activity of bimetallic zinc oxide and silver/zinc oxide nanoparticles

**DOI:** 10.1016/j.heliyon.2024.e31330

**Published:** 2024-05-15

**Authors:** Augustine Innalegwu Daniel, Enriquay Smith, Ali Al-Hashimi, Arun Gokul, Marshall Keyster, Ashwil Klein

**Affiliations:** a Plant Omics Laboratory, Department of Biotechnology, Faculty of Natural Sciences, University of the Western Cape, Robert Sobukwe Road, Bellville, 7535, South Africa; b Department of Biochemistry, School of Life Sciences, Federal University of Technology, P.M.B 65, Minna, Niger State, Nigeria; c Department of Plant Sciences, Qwaqwa Campus, University of the Free State, Phuthadithjaba, 9866, South Africa; d Environmental Biotechnology Laboratory, Department of Biotechnology, Faculty of Natural Sciences, University of the Western Cape, Robert Sobukwe Road, Bellville, 7535, South Africa

**Keywords:** *Alternaria alternata*, Biological control, Biosynthesis, Nanofungicide, Plant pathogens, Silver nitrate zinc oxide

## Abstract

*Alternaria alternata* is an opportunistic phytopathogen that negatively impact the growth and production of a wide variety of host plants. In this study, we evaluated the antifungal potential of biogenic ZnO, and bimetallic silver and zinc oxide (Ag/ZnO) nanoparticles synthesized using seed extract of *Abrus precatorious* and characterized using different analytical tools. *In vitro* antifungal potentials of ZnO and Ag/ZnO nanoparticles were carried out using the food poison technique. Morphological and ultrastructure of the *A. alternata* treated with the nanoparticles were carried out using high resolution scanning and transmission electron microscopy (HRSEM and HRTEM). In addition, changes in polysaccharide production, chitin content and enzymatic (cellulase and lipase) activities of *A. alternata* were assayed. Double peak signifying a UV_max_ of 353.88 and 417.25 nm representing Ag and ZnO respectively was formed in the bimetallic nanoparticles. HRSEM and HRTEM results shows agglomerated nanoparticles with particle and crystallite size of 23.94 and 16.84 nm for ZnO nanoparticles, 35.12 and 28.99 nm for Ag/ZnO nanoparticles respectively. *In vitro* antifungal assay shows a significant concentration-dependent inhibition (p < 0.05) of *A. alternata* mycelia with highest percentage inhibition of 73.93 % (ZnO nanoparticles) and 68.26 % (Ag/ZnO nanoparticles) at 200 ppm. HRSEM and HRTEM micrographs of the treated *A. alternata* mycelia shows alteration of the cellular structure, clearance of the cytoplasmic organelles and localization of the nanoparticles within the cell. *A. alternata* treated with 200 ppm nanoparticles show a significant decrease (p < 0.05) in the polysaccharides and chitin contents, cellulase and lipase activities. The results suggests that ZnO and Ag/ZnO nanoparticles mode of action may be via alteration of the fungal cell wall through the inhibition of polysaccharides, chitin, cellulases and lipases synthesis. ZnO and Ag/ZnO nanoparticles may be a promising tool for the management and control of disease causing fungal phytopathogens.

## 1 Introduction

Fungal diseases account for over 80 % annual food crop losses, contributing to high food insecurity globally [1]. *Alternaria alternata* has been reported to cause serious damage to crop plants by affecting the leaves and fruits pre- and post-harvest resulting in over 60 % damage [2,3]. The symptoms of the disease caused by *A. alternata* appear as tiny, rounded, brown spots on leaves and fruits. As the disease progress, these spots may take on an irregular shape and ultimately covering a major part of the leaves, causing them to wither, dry out, and fall off. On the seed, this disease causes decay, which increases the risk of mycotoxin production by the fungi [4]. The disease is a major risk to seed quality pre- and post-harvest [5].

Different control strategies such as chemical, biological, and physical methods have been explored to control *A. alternata*. Chemical control using fungicides is regarded as the most efficient strategy to control *A. alternata*. However, excessive use of chemical fungicides has led to downstream side effects including the emergence of resistant pathogen strains, soil acidification, groundwater contamination, and destruction of ecosystems which can negatively impact human and animal health [1,6]. An alternative to chemical control is the use of biocontrol agents, which is a suitable substitute for these fungicides [7]. Maintaining hygiene standards and inconsistent application are problems associated with employing microbes as biocontrol agents. The need to test host specificity over an extended period, inability to completely eradicate pest, cost of field survey and early testing phases are all factors that affect the use of biological control agents [8]. Therefore, there is need to employ cost-effective and sustainable alternatives such as nanotechnology to control plant pathogens such as *A. alternata* [6].

The use of nanotechnology in agriculture for food production and the development of nanopesticides and nanofertilizers has been well documented [6,9,10]. Metallic nanoparticles made of metal or non-metallic oxides could serve as alternative to the chemical control strategies. Biologically derived nanoparticles have emerged as a promising tool for controlling disease causing pathogens in food crops due to their low toxicity profile [7].

Zinc oxide (ZnO) nanoparticles is a widely nanomaterials with application in food and pharmaceutical industries [11]. This nanoparticle is used for drug delivery, topical treatments for skin diseases and dietary additives [12]. ZnO nanoparticles have been reported to significantly inhibit microbial growth [13]. According to literature, the bioactivity and biological applications of ZnO nanoparticles can be enhanced when present in a composite-based formulations with other metals, none metals or compounds. These formulations consist of nanostructured ZnO coupled with other metallic or metal oxide nanoparticles as well as antibiotics or biocompatible components derived from biological sources [11]. For example, it was reported that the antibacterial efficacy ZnO and silver (Ag) nanoparticles was enhanced when combined with TiO_2_ and chitosan [14,15]. Furthermore, ZnO nanoparticles combined with β-lactam antibiotics have demonstrated improved synergistic bioactivity [11]. Therefore, the aim of this study was to evaluate the antifungal potential of biogenic ZnO and bimetallic Ag/ZnO nanoparticles against *A. alternata* isolated from diseased wheat plants. The objective of this study are (i) to isolate and characterize *A. alternata* from diseased wheat plants, (ii) to synthesize, and characterize ZnO and bimetallic Ag/ZnO nanoparticles using aqueous extract of *Abrus precatorious* seed, (iii) to evaluate the *in vitro* anti-alternaria activity of ZnO and bimetallic Ag/ZnO nanoparticles, (iv) to study the effects of the nanoparticles on the ultra-structure of *A. alternata* using scanning and transmission electron microscopy and (v) to determine the effect of the nanoparticles on the biochemical and enzymatic activities of *A. alternata*.

## 2 Material and methods

### 2.1 Fungal isolation

The strain of *Alternaria alternata* BBS-CT21 used in this study was sourced from diseased wheat plant roots obtained from Cape Farms, South Africa (33°48′26.6″S 18°33′45.9″E) in 2022. To ensure purity, the fungal strain was cultured on potato dextrose agar (PDA) from Sigma-Aldrich, Germany, at 28 °C for a period of 7 days. Subsequently, the cultures were preserved at 4 °C until required for subsequent experiments.

### 2.2 DNA extraction and sequence identification

The DNA extraction was carried out using Zymo Research Quick-DNA Fungal-Bacterial Miniprep kit (Catalogue Number D6005, Zymo Research) following the manufacturer instruction. Amplification of the DNA extract and phylogenetic analysis was carried out following the protocol outlined by Daniel et al. [16].

### 2.3 Preparation of plant extract

The seeds of *Abrus precatorius* were crushed into fine powder using a metallic blade kitchen blender. Twenty grams (20 g) of *A. precatorius* powder was extracted with 400 mL of distilled water at 45 °C for 30 min with continuous stirring using magnetic stirrer. The extract was filter paper to obtain a fine filtrate which was stored at 4 °C for subsequent usage.

### 2.4 Green synthesis of zinc oxide nanoparticles

The extract of *A. precatorius* (20 mL) was mixed with 100 mL 0.2 M solution of ZnSO_4_. The reaction pH was adjusted to 9 using 0.2 M NaOH and stirred continuously at 60 °C for 2 h. The synthesis of the nanoparticle confirmed by a change in color from yellow to light green. The obtained precipitate was separated using centrifugation at 9000×*g* rpm for 10 min and further washed with reverse osmosis water (Milli-Q) to remove any trace impurities. Finally, the nanoparticles was calcined at 400 °C for 3 h in a muffle furnace to fine white powder.

### 2.5 Green synthesis of bimetallic silver/zinc oxide (Ag/ZnO) nanoparticles

For the synthesis of Bimetallic Ag/ZnO nanoparticles, 50 mL each of 0.2 M AgNO_3_ and ZnSO_4_ solutions were mixed and stirred for 30 min to achieve homogeneity. Seed extract of *A. precatorius* (20 mL) was added to the above solution and the pH was adjusted to 9 using 0.2 M NaOH. The mixture was then stirred continuously for 2 h at 60 °C. The synthesis of the nanoparticle confirmed by a color change from black to reddish-brown. The obtained precipitate was separated using centrifugation at 9000×*g* rpm for 10 min and further washed with reverse osmosis water (Milli-Q) to remove any trace impurities. Finally, the nanoparticles was calcined at 400 °C for 3 h in a muffle furnace to fine white powder.

### 2.6 Characterization of nanoparticles

Various analytical techniques were used to characterize the synthesized nanoparticles. The absorption spectrum of the nanoparticles was determined using a double-beam UV–visible spectrophotometer. The chemical composition of the nanoparticles was determined using Energy-Dispersive X-ray spectroscopy (EDX) while High-Resolution Transmission and Scanning Electron Microscopy (HRTEM and HRSEM) were used to examine the shape and morphology of the nanoparticles. Additionally, X-ray Diffractometry (XRD) was used to determine the dispersity and crystallinity of the nanoparticles.

### 2.7 Determination of anti-alternaria activity of ZnO and bimetallic Ag/ZnO nanoparticles

The antifungal activity of ZnO and bimetallic Ag/ZnO nanoparticles was determined using the modified poisoned food technique method described by Adjou Euloge et al. [17] and Gakuubi et al. [18] as reported by Daniel et al. [16].

### 2.8 Effect of ZnO and bimetallic Ag/ZnO nanoparticles on mycelial morphology and ultrastructure of *A. alternata*

The effect of ZnO and bimetallic Ag/ZnO nanoparticles on the morphological and ultrastructural alterations of *A. alternata* was determined using HRSEM and HRTEM techniques as reported by Dhiman et al. [7] and Daniel et al. [16].

### 2.9 Biochemical analysis of *A. alternata* treated with ZnO and bimetallic Ag/ZnO nanoparticles

For biochemical analysis, *A. alternata* was cultivated in potato dextrose broth (PDB) supplemented with 200 ppm of either ZnO or bimetallic Ag/ZnO nanoparticles for a duration of 10 days. Depending on the specific assay, either fungal biomass or filtrate was utilized for the analysis.

#### 2.9.1 Estimation of extracellular and intracellular polysaccharides contents

Extracellular and intracellular polysaccharides contents of fungal mycelia treated with the nanoparticles according to a modified protocol reported by Dhiman et al. [7]. The amount of intracellular extracellular polysaccharides was quantified using Anthrone method.

#### 2.9.2 Estimation of chitin content

The protocol reported by Ospina Álvarez et al. [19] was used to estimate the chitin contents of *A. alternata* treated with the nanoparticles. The amount of the chitin was expressed and mg/g of fresh mycelia weight.

#### 2.9.3 Cellulase activity

Endoglucanase and exoglucanase activity of *A. alternata* was determined using a modified Mendel's media as reported by Daniel et al. [16]. Endoglucanase and exoglucanase activities were estimated using the di-nitrosalicylic acid (DNS) method [20]. The enzyme activity was expressed as unit per millilitre per minute (U/mL/min). One unit of enzyme is defined as the amount of enzyme which releases 1 mL of fatty acid per minute under specified assay conditions.

#### 2.9.4 Lipase activity

Lipase activity of *A. alternata* treated with ZnO and bimetallic Ag/ZnO was determined according to the reported protocol by Iftikhar et al. [21]. The enzyme activity was expressed as unit per millilitre per minute (U/mL/min). One unit of enzyme is defined as the amount of enzyme which releases 1 mL of fatty acid per minute under specified assay conditions.

### 2.10 Statistical analysis

The data obtained from this study were statistical analyzed using GraphPad Prism version 8.0.1 and SPSS version 26. Results were presented as mean ± standard error of mean (SEM) from triplicate measurements. Significance levels were determined using one-way analysis of variance (ANOVA), and differences were considered significant at p < 0.05.

## 3 Results and discussion

### 3.1 Molecular identification and phylogenetic analysis of the fungal isolate

**Fig. 1:**
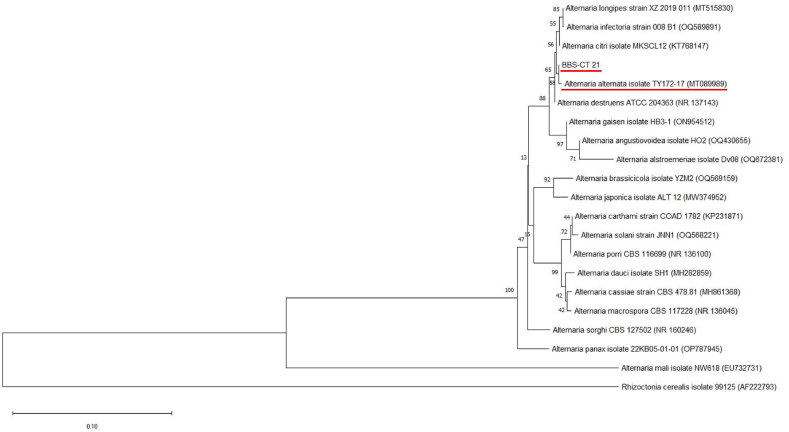
Phylogenetic analysis of ITS rDNA sequences of isolate BBS-CT 21 with reference sequences from NCBI (Table 1) using the MEGA maximum likelihood method based on the Tamura-Nei model. The bootstrap values are expressed as a percentage of 500 replicates. *Rhizoctonia cerealis* isolate 99125 (AF222793) was used as outgroup. The identified fungal isolate and its closest hit are underlined in red.

**Fig. 2:**
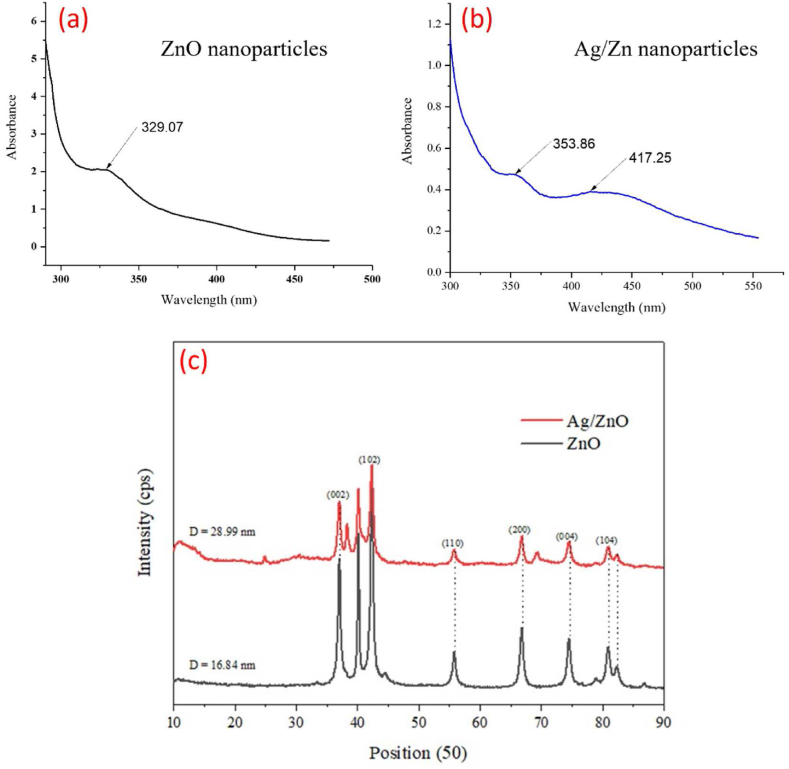
UV–vis absorption spectra (a–b) and XRD plot (c) of biogenic ZnO nanoparticles and bimetallic Ag/ZnO nanoparticle synthesized using seed extract of *A. precatorious.*

The DNA sequence of the fungi isolate was blasted using the basic local alignment search tool (BLAST) against the non-redundant NCBI database. It was discovered that the fungi isolate shared a significant degree of sequence similarity with *Alternaria alternata* (Table 1). The isolate (BBS-CT 21) has a close phylogenetic relationship with *Alternaria alternata* (Fig. 2). A BLAST search against NCBI database revealed that isolate BBS-CT 21 and isolate TY172-17 of *Alternaria alternata* are 100 % identical (Table 1). The phylogenetic link between the discovered isolate and certain database sequences was ascertained using the greatest likelihood method (Fig. 1).

**Table 1:** Identification of fungal isolate based on ITS rDNA sequencing.

Fungal Isolate	Host	Tissue	Top BLAST Hit	Accession Number	Sequence Identity (%)
BBS-CT 21	*Triticum aestivum*	leaves	*Alternaria alternata isolate TY172-17*	MT089989.1	100.00

### 3.2 Characterization of biosynthesized ZnO and bimetallic Ag/ZnO nanoparticles

#### 3.2.1 UV–visible spectrophotometric and XRD analysis of biogenic ZnO and bimetallic Ag/ZnO nanoparticles

The UV–visible spectra absorption of the synthesized nanoparticles shows that ZnO nanoparticles absorbed maximally at 329.07 nm (Fig. 2a) while bimetallic Ag/ZnO shows the presence of two absorption peaks at 353.86 and 417.25 nm representing ZnO and Ag nanoparticles respectively (Fig. 2b).

X-Ray diffraction (XRD) analysis of ZnO nanoparticles revealed a hexagonal structure with peaks at 2θ values of 37.15°, 42.24°, 56.90°, 66.85°, 74.66° and 80.91 with a diffraction plane of (002), (102), (110), (200), (004) and (104) which matches a JCP2 file number 36–1451 (Fig. 2c). The bimetallic (Ag/ZnO) nanoparticles show the presence of peaks similar to Ag and ZnO nanoparticles with some shifts and the emergence of some unaccounted peaks (Fig. 2c). This could be due to the interaction between the two metals resulting in orbital vibrations and adjustment or shift in peaks. The crystallite size of the nanoparticles was calculated using the Debye Scherrer equation: $D=\frac{K\lambda }{\beta \text{Cos}\theta }$ where D is the nanoparticles crystalline size, K represents the Scherrer constant (0.98), λ denotes the wavelength (1.54), β denotes the full width at half maximum (FWHM). The average crystallite size (D) of ZnO and bimetallic Ag/ZnO nanoparticles are 16.84 and 28.99 nm respectively (Fig. 2c).

#### 3.2.2 HRSEM, HRTEM and particle size of biogenic ZnO and bimetallic Ag/Zn nanoparticles

**Fig. 3:**
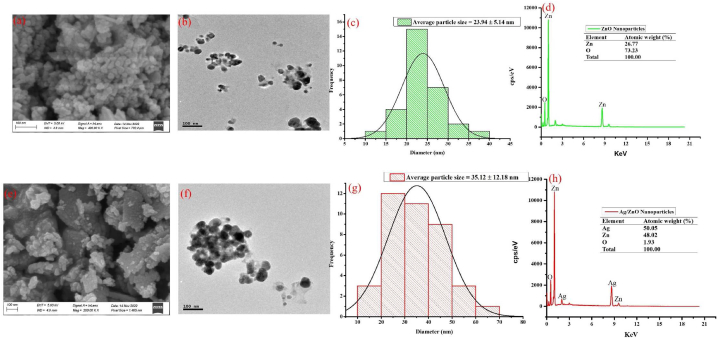
HRSEM, HRTEM, particle size distribution and EDX analysis of biogenic ZnO (a–d) and bimetallic Ag/ZnO (e–h) nanoparticles synthesized using seed extract of *A. precatorious.*

HRSEM and HRTEM analysis of ZnO and bimetallic Ag/ZnO nanoparticles (Fig. 3) revealed a cubical and monodispersed crystal grains of ZnO nanoparticles while bimetallic Ag/ZnO nanoparticles shows spherical and agglomerated particles (Fig. 3a, b, e and f). The agglomeration in bimetallic Ag/ZnO nanoparticles also shows the interaction between the two metals resulting in vibrational nuclear pull of electrons in their orbitals. The particle size distribution of the nanoparticles was calculated from the HRTEM micrograph and the result shows that ZnO nanoparticles have a particle size of 23.94 ± 5.14 nm while bimetallic Ag/ZnO nanoparticles have a particle size of 35.12 ± 12.18 nm (Fig. 3c and g).

#### 3.2.3 Elemental composition of biogenic ZnO and bimetallic Ag/ZnO nanoparticles

The elemental composition of the biogenic nanoparticles was carried out to determine the constituent elements present in the nanoparticles. The result shows the presence of Ag and Zn in various amount with ZnO nanoparticles containing Zn and O at a ratio of 3:1 (Fig. 3d) while bimetallic Ag and ZnO nanoparticles contains Ag and Zn at an approximately equal amount (1:1) with a trace of oxygen (1.93 %) (Fig. 3h). Finally, there was an emission of Zn and O at an energy level of 1.24 and 0.71 KeV respectively and 2.21 KeV for Ag which have been reported to be characteristics of the binding energy of Zn, O and Ag respectively (Fig. 3d).

### 3.3 *In vitro* antifungal activity of ZnO and bimetallic Ag/ZnO nanoparticles against *A. alternata*

**Fig. 4:**
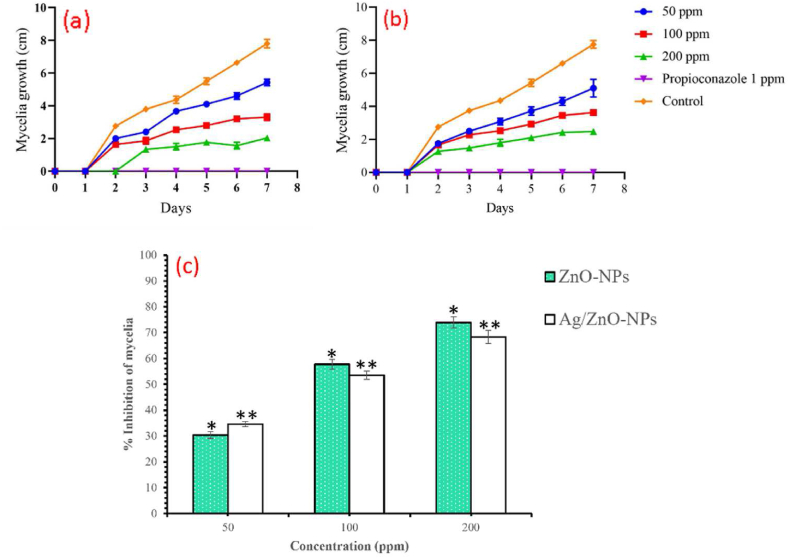
The growth rate of *A. alternaria* in response to propiconazole and ZnO (a) and bimetallic Ag/ZnO (b) nanoparticles. The concentration dependent inhibition of *A. alternaria* in response to ZnO and bimetallic Ag/ZnO nanoparticles (c).

The growth rate (in cm) of *A. alternata* mycelia was measured for a period of 7 days in response to ZnO and bimetallic Ag/ZnO nanoparticles treatment and propiconazole (a commercial antifungal compound) used as positive control (Fig. 4 a and b). The results show a progressive growth of the mycelia in the control plate to about 8 cm in diameter compared to fungi treated with 200 ppm of ZnO an bimetallic Ag/ZnO nanoparticles which shows a significant inhibition (p < 0.05) of their growth within the 7-day period. In addition, the mycelia growth inhibition of *A. alternata* was also measured in response to the nanoparticle's treatment (Fig. 4c). The antifungal activity of ZnO and bimetallic Ag/ZnO nanoparticles shows the inhibition of the fungal mycelia in a dose-dependent manner with ZnO nanoparticles showing highest inhibition (78.93 %) at 200 ppm while bimetallic Ag/ZnO nanoparticles caused an inhibition of 68.27 % (Fig. 4c) compared to the standard antifungal compound (1 ppm propiconazole) which shows complete inhibition of *A. alternaria*.

### 3.4 Effects of biogenic ZnO and bimetallic Ag/ZnO nanoparticles on the ultrastructure and morphology of *A. alternata*

**Fig. 5:**
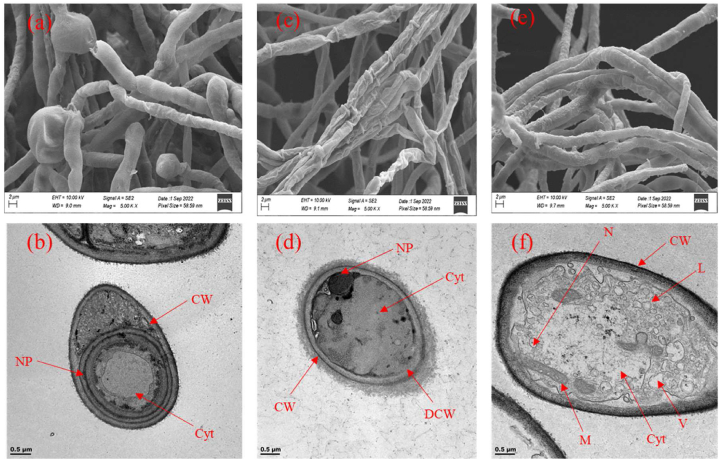
HRSEM and HRTEM images of *A. alternaria* mycelia treated with 200 ppm ZnO (a and b), bimetallic Ag/ZnO (c and d) nanoparticles and untreated control (e and f). Keys: CW = cell wall, DCW = distorted cell wall, Cyt = cytoplasm, N = nucleus, NP = nanoparticles, L = lipid droplets, M = mitochondria, V = vacuole.

The effects of biogenic ZnO and bimetallic Ag/ZnO nanoparticles on the ultrastructure and morphology of *A. alternata* was observed under SEM and TEM. Under SEM, *A. alternata* mycelia treated with 200 ppm of ZnO and bimetallic Ag/ZnO nanoparticles (Fig. 5a–c and e) shows a change in the morphological structure of the organism with ZnO causing an inflammation or swelling of the mycelia (Fig. 5a) while bimetallic Ag/ZnO nanoparticles causes alteration such as twisting with shrunken, shrived and breakage in the morphology of the fungal mycelia (Fig. 5c) when compared with the control which shows normal mycelia structure (Fig. 5e). Under transmission electron microscope (Fig. 5b–d and f), ZnO nanoparticles shows complete clearance of the cellular organelles and a reduction in the thickness of cell wall (Fig. 5b) while the bimetallic Ag/ZnO nanoparticles caused severe cellular damage, including clearance of the cytoplasmic organelles, vacuolation and alteration or clearance of the cell wall (Fig. 5d). The control treatment structure was unaltered, with a smooth cell wall and the presence of cytoplasmic organelles (Fig. 5f).

### 3.5 Effects of biogenic ZnO and bimetallic Ag/ZnO nanoparticle treatment on polysaccharides and chitin contents

**Fig. 6:**
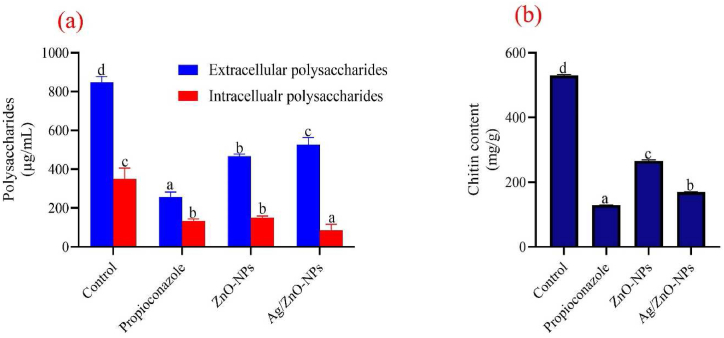
Effect of ZnO and Ag/ZnO nanoparticles on (a) intracellular and extracellular polysaccharide and (b) chitin contents of *A. alternata*.

The effect of the nanoparticle's treatment on the biochemical and enzyme activities of the fungi was also assayed in this study (Fig. 6). Fig. 6a shows the effect of ZnO and bimetallic Ag/ZnO nanoparticle treatment on the intracellular and extracellular polysaccharides contents of *A. alternata*. Higher intracellular polysaccharide content (350.81 ± 5.53 μg/mL) was observed in the control group with a significant reduction (p < 0.05) in ZnO nanoparticles treatment group (149.68 ± 9.03 μg/mL), followed by propiconazole (133.95 ± 9.48 μg/mL) while bimetallic Ag/ZnO nanoparticles has the least (86.13 ± 3.05 μg/mL) intracellular polysaccharide content. Analysis of the extracellular polysaccharide contents shows 847.74 ± 2.9, 257.98 ± 2.48, 467.10 ± 12.07, and 526.61 ± 3.70 μg/mL in the control, propiconazole, ZnO and bimetallic Ag/ZnO nanoparticles treatment respectively. The chitin content in the control group (530.20 ± 2.55 mg/g) was significantly higher (p < 0.05) followed by ZnO nanoparticles (266.30 ± 2.97 mg/g), bimetallic Ag/ZnO nanoparticles (169.40 ± 1.41 mg/g) while group treated with propiconazole has the least chitin content (129.90 ± 01.4 mg/g) (Fig. 6b).

### 3.6 Effects of ZnO and bimetallic Ag/ZnO nanoparticles on cellulase and lipase activity

**Fig. 7:**
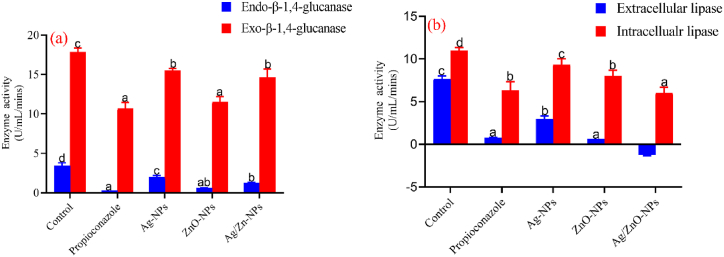
Effects of ZnO and bimetallic Ag/ZnO treatments on (a) endo-β-1,4-glucanase and exo-β-1,4-glucanase and (b) extracellular and intracellular lipase of *A. alternata*.

Changes in *A. alternata* exoglucanase and endoglucanase activity was evaluated after treatment (for 10 days) with ZnO and bimetallic Ag/ZnO nanoparticles (Fig. 7a). Relative to the control treatment (3.46 ± 0.23 U/mL/h), there was a significant decrease (p < 0.05) in endoglucanase activity in all treatments with the highest decrease observed in fungi treated with propiconazole (0.31 ± 0.00 U/mL/h) followed by ZnO (0.62 ± 0.10 U/mL/h) and Ag/ZnO (1.27 ± 0.04 U/mL/h) nanoparticles (Fig. 7a). A similar trend was observed for exoglucanase activity in response to the same treatments. Exoglucanase activity in the control treatment (17.89 ± 0.34 U/mL/h) was significantly higher (p < 0.05), followed by Ag/ZnO (14.62 ± 0.77 U/mL/h), ZnO (11.54 ± 0.46 U/mL/h) and propiconazole (10.69 ± 0.54 U/mL/h) treatments (Fig. 7a). This shows the inhibition of the enzyme in the nanoparticles and propiconazole treatment compared to the control group.

Furthermore, the intracellular lipase activity was significantly lower in Ag/ZnO nanoparticles (6.00 ± 0.67 U/mL/h) followed by propiconazole (6.33 ± 1.00 U/mL/h) and ZnO nanoparticles (8.01 ± 0.67 U/mL/h) when compared to the untreated control (11.00 ± 0.33 U/mL/h) (Fig. 7b).

## 4 Discussion

The synthesis of materials with the best physical and chemical properties for the management and control of microbial infections is gaining more attention in composite-based nanotechnology [11,14]. Metallic oxide nanoparticles including ZnO nanoparticles, have already been extensively use in the field of nanomedicine [12,22]. The pharmacological activity of Ag nanoparticles in biomedical applications is also well-documented [23,24]. Therefore, in this study we evaluated the antifungal potential of biogenic ZnO and bimetallic Ag/ZnO nanoparticles against *Alternaria alternata*. Biogenic or green synthesis is the most preferred method of synthesis of nanomaterials because of its advantages such as eco friendliness, ease of synthesis and cost effectiveness over other methods of synthesis [25,26]. The synthesis of ZnO and bimetallic Ag/ZnO nanoparticles in this study was characterized using different analytical techniques. UV–visible spectrophotometric analysis is used as a preliminary screen to confirm the synthesis of any nanomaterials since each nanomaterials has a unique UV range where it absorbs maximally. Spectrophotometric scanning of ZnO nanoparticles shows a maximum UV absorption in the region of 329.07 nm (Fig. 2a) while bimetallic Ag/ZnO nanoparticles shows the presence of two distinct peaks at 353.86 and 417.25 nm (Fig. 2b). Previous study by Corciova et al. [26] reported that Ag nanoparticles have a maximum UV absorption around 400–450 nm while Kumar et al. [11] reported that ZnO nanoparticles absorbed maximally within 300–350 nm. The presences of these peaks in the bimetallic Ag/ZnO therefore confirmed that the nanoparticles consist of these metals. The appearance of these peaks within these UV region may be attributed to the excitation of the surface plasmon resonance caused by the two metals resulting in orbital vibrations or shift which may lead to the formation of the nanoparticles hence affecting the physical, chemical, and optical properties of the nanoparticles [11,26]. XRD data from this study shows that the ZnO and bimetallic Ag/ZnO nanoparticles have an average crystallite size (D) of 16.84 and 28.99 nm respectively (Fig. 2c). The morphology and shape of the synthesized nanoparticles was determined using HRSEM and HRTEM which shows a cubical and spherical nature of Ag and ZnO nanoparticles (Fig. 3a and b, 3e and 3f). The particle sizes of the nanoparticles calculated from the TEM image shows that both ZnO and bimetallic Ag/ZnO nanoparticles particle sizes are <40 nm (Fig. 3c and g). Particle size is of great importance in the field of nanotechnology because the aim is to synthesize nanomaterials with higher surface area with improve biological, physical, and chemical properties. The effects of crystallite and particle sizes on the physical and magneto-resistive and biological properties of properties of nanomaterials are matters of great importance [27]. According to Malandrakis et al. [28], Pillai et al. [29], and Pariona et al. [30], antifungal activities of nanoparticles majorly depends on their particle size. Smaller particle and crystallite sizes (<60 nm) nanoparticles are more toxic to fungi cell than nanoparticles with larger sizes (>100 nm) [27]. Furthermore, the elemental composition of the nanoparticles was determine using Energy-Dispersive X-Ray Spectroscopy (EDX) (Fig. 3d and h). The results shows the presence of the constituent elements in ZnO nanoparticle at equal proportion (Fig. 3d) with an emission at 1.24 and 0.71 KeV for Zn and O respectively which are characteristics of the binding energies of Zn and O [31]. A trace of oxygen in bimetallic Ag/ZnO nanoparticle was reported with a binding energy of 0.76 KeV while Zn and Ag were emitted at 1.26 and 2.21 KeV respectively representing the binding energy of Zn and Ag (Fig. 3h) [31,32]. The result also shows the presence of some unaccounted peaks which could be due to the presence of impurities from the plant extracts, or the various salts used in the synthesis of the nanoparticles. The essence of characterizing the nanomaterials is to ensure the purity of the nanoparticles and to also ensure that the biological activity of the nanoparticles is not affected by contaminant present in the nanoparticles [[31], [32], [33]]. Therefore, in this study we confirmed that the nanoparticles synthesized using seed extract of *A. precatorious* was effectively carried out and the nanomaterials contains Ag, Zn and O in different ratios in bimetallic Ag/ZnO nanoparticles while ZnO nanoparticles contain only Zn and O as the major elements.

*In vitro* antifungal activity of ZnO and bimetallic Ag/ZnO nanoparticles against *A. alternata* shows the inhibition of the fungal mycelia in a concentration-dependant manner in both ZnO and bimetallic Ag/ZnO nanoparticles (Fig. 4a–c). Different studies have reported on the antifungal activities of Ag and ZnO nanoparticles against different pathogenic fungi [2,6,7,26,29,30,[34], [35], [36], [37], [38], [39]]. Furthermore, the findings from this study also agrees with previous reports by Dhiman et al. [33] and Kumar et al. [11] who independently reported on the antifungal potential of ZnO nanoparticles against *Alternaria brassicae, Penicillium funiculosum, Aspergillus niger and Fusarium oxysporum* and *Candida albican.* Their report shows that ZnO nanoparticles significantly inhibit these fungi pathogens by inhibiting their growth. The antifungal activity of bimetallic Ag/ZnO nanoparticles maybe due to the synergistic presence of Ag and Zn in a composite form. Different literatures have reported on the antifungal activity of these respective metals such as Bahrami-Teimoori et al. [35] Ouda et al. [38] who independently reported on the significant inhibition of *A*. *alternata* by Ag nanoparticles and Paraguay-Delgado et al. [40] and Zhu et al. [39] who reported on the antifungal activities of ZnO nanoparticles against pathogenic plant fungi. However, limited evidence exist in literatures on the antimicrobial activity of bimetallic Ag/ZnO nanoparticles [14]. According to Malandrakis et al. [41], ZnO nanoparticles are more toxic against fungi than Ag nanoparticles. In our previous study, Ag nanoparticles was reported to inhibit about 54.61 % growth of *A. alternata* mycelia [16]. Therefore, this study shows that the combination of these metals in a composite form have enhanced the antifungal activity of the nanoparticles which agrees with the report of Kumar et al. [11] and Ye et al. [15] that the antimicrobial activity of metallic nanoparticles can be enhance by combining them with other metallic nanoparticles in a composite form. Propioconazole in comparison to the nanoparticles shows a complete inhibition the fungal mycelia growth. This agrees with the report of Zhang et al. [42] who tested the antifungal activity of 0.1 ppm of propioconazole against *Penicillium digitatum* and reported that the fungicide causes over 80 % inhibition of the fungi.

To support the *in vitro* anti-alternaria activity of the nanoparticles, we determine the effect of the nanoparticles on the ultra-structure and morphology of the fungi (Fig. 5a–d). Previous cytological reports shows that nanoparticles can severely damage the cellular components of fungi cell walls, destroyed the structure and integrity of cell membranes resulting in wrinkled and depressed spores and mycelia [43,44]. These effects can result in less oxygen consumption by the fungal mycelia because of damage respiratory chain due to inactivation of important proteins or enzymes and bound lipids leading to enzymes induced cell lysis. The effects of the nanoparticles on the fungi structure will eventually lead to twisting and expansion of the cell until they are shrunken and died [43,44]. In this study, treatment of *A. alternata* with ZnO and bimetallic Ag/ZnO nanoparticles increased the permeability of the fungal cell membranes and compromised their integrity which may allow extra water and the nanoparticles to permeate the cells causing the volume of the cells to enlarge under the influence of osmotic pressure and ultimately resulting in the leakage of the cytoplasmic contents of the cell. SEM image of the treated fungi shows that ZnO and bimetallic Ag/ZnO nanoparticle treatment resulted in twisted, shrunken, and shrivelled hyphae of the fungi (Fig. 5a and c). The stress symptoms were more severe in the bimetallic Ag/ZnO nanoparticles than ZnO nanoparticles treatment. Furthermore, TEM image shows that ZnO and bimetallic Ag/ZnO nanoparticles treatment substantially altered the cellular structure and organelles of the fungi (Fig. 5b and d), which was consistent with morphological observation under SEM. According to Zhang et al. [42], propiconazole significantly increased reactive oxygen species (ROS) levels of germinating conidia of fungi, which agrees with the theory of electron transport process. The increased level of ROS may also be the conventionally accepted mode of action of demethylation inhibitor (DMI) fungicides. Inhibition of ergosterol biosynthesis will lead to the disruption of the plasma membrane, which will damage the normal function of organelles. TEM image in the present study shows the deformed nature of the fungi mitochondria treated with the nanoparticles compared with the control treatment (Fig. 5b–d and f) which may lead to increased ROS generation in the fungi [45].

In the design of any antimicrobial agents, it is always important to produce materials that will alter the normal cell cycle of the organism to prevent their growth and replications. The effect of the nanoparticle's treatment on the biochemical and enzyme activities of the fungi was also analyzed in this study. Most antimicrobial agents target the cell wall or other enzymatic processes that are essential for the survival of the microorganisms [[46], [47], [48]]. Chitin and polysaccharides serve as the fundamental building blocks of the fungal cell wall and provide the cell with its shape and rigidity [7]. For the cell's immediate energy requirements, polysaccharides, also known as glucans, are carbohydrates that are abundant in fungi and can be broken down by enzymes such as glucanase into glucose which can be use by the organism. From this study, extracellular and intracellular polysaccharides contents of the fungi significantly decreased (p < 0.05) when compared to the control treatment (Fig. 6a), which can be attributed to the nanoparticle treatment's effects. The chitin content of *A. alternata* (Fig. 6b) shows a significant decrease (p < 0.05) in response to ZnO and bimetallic Ag/ZnO nanoparticles treatment compared to the control, demonstrating the effect of the nanoparticles to inhibit chitin synthesis in the fungi. This shows that ZnO and bimetallic Ag/ZnO nanoparticles were toxic to *A. alternata* cell imposing stress on the fungi cell*.* Furthermore, according to Dhiman et al. [7], increase in ZnO nanoparticles concentration affect the level chitin present in the cell wall of the fungi. Chitin, a crucial component of fungal cell walls, correlates with the level of stress endured by fungi. Elevated chitin levels reinforce cell walls, enhancing their resilience against environmental pressure. Following cell wall damage, class IV enzymes, notably ScChs3 and CaChs3, often overproduce chitin in response [49]. Interfering with chitin synthesis leads to deformities and osmotic instability in fungal cells [50]. Chitin and polysaccharides serves various functions, such as maintaining cell shape, protecting fungal cells from external stresses, chemical absorption, adhesion, signal transmission, and producing cell wall components [7]. Therefore, a decrease in the polysaccharides and chitin contents of the fungi is an indication of the nanoparticle's toxicity to the fungi cell.

Various microorganisms, such as fungi, bacteria, and archaea, generate cellulases, a group of enzymes employed to break down cellulose into glucose and other fermentable sugars [51]. Endoglucanases cleave β-1,4-glucan linkages within cellulose molecules without specific directionality, while β-glucosidases break down disaccharides to liberate glucose units. Exoglucanases, on the other hand, target both the reducing and non-reducing ends of cellulose, removing cellobiose fragments [52]. Fig. 7a shows the effects of ZnO and bimetallic Ag/ZnO nanoparticles on exo-β-1,4-glucanase and endo-β-1,4-glucanase activity of *A. alternata*. The result shows a significant decrease (p < 0.05) in the activities of the enzymes compared to the control (p < 0.05).

Lipases are group of enzymes present in fungi which has been reported to contribute to fungi pathogenesis [53,54]. Extracellular lipase has been proposed to be the potential virulence factors of pathogenic fungi which is used to directly cause damage to host cells by physical forces during host cell invasion or escape which could potentially degrade components of the cuticle [53], and also through hydrolytic enzymes (like proteases or lipases) or lytictoxins in the form of peptides or small metabolites [54]. From this study, the activities of lipase enzymes (extracellular and intracellular) in the fungi treated with ZnO and bimetallic Ag/ZnO nanoparticles were significant decreased (Fig. 7b). This can also be attributed to the toxicity of the nanoparticles which may inhibit the production of these enzymes hence reducing their virulence and their ability to invade their hosts' tissues by hydrolysing the cellulose using these enzymes. The report of this study also agrees with the report of Jasim [55]. In his report, ZnO nanoparticles treatment significantly reduced the activity of lipase enzymes in *Aspergillus fumigatus* and *Candida albicans*.

## 5 Conclusion

In conclusion, the result of this study shows that ZnO and bimetallic Ag/ZnO nanoparticles synthesized using seed extract of *A. precatorious* significantly inhibit the mycelia and growth of *A. alternata*. Furthermore, based on SEM and TEM image, the nanoparticles significantly alter the morphology and anatomical structure of *A. alternata* resulting in clearing of the cell walls and cellular organelles. The nanoparticles also cause a reduction in the polysaccharides and chitin contents and membrane associated enzymatic activities (lipase, cellulase and glucanase) of *A. alternata*. Based on this outcome, ZnO and bimetallic Ag/ZnO nanoparticles may be considered good candidates for the development of novel antifungal compounds to combat plant disease-causing pathogens. In other to deduce the overall anti-alternaria activity mode of action of the nanoparticles, we proposed that further studies should be carried out on the effect of the nanoparticles on the antioxidant system and proteome profile of *A. alternata*.

## Data availability statement

Not applicable.

## CRediT authorship contribution statement

**Augustine Innalegwu Daniel:** Writing – review & editing, Writing – original draft, Visualization, Validation, Software, Methodology, Investigation, Formal analysis, Data curation, Conceptualization. **Enriquay Smith:** Writing – review & editing, Formal analysis. **Ali Al-Hashimi:** Writing – review & editing, Writing – original draft, Formal analysis, Data curation. **Arun Gokul:** Writing – review & editing, Formal analysis. **Marshall Keyster:** Writing – review & editing, Supervision, Conceptualization. **Ashwil Klein:** Writing – review & editing, Writing – original draft, Supervision, Project administration, Funding acquisition, Conceptualization.

## Declaration of competing interest

The authors declare the following financial interests/personal relationships which may be considered as potential competing interests:

Ashwil Klein reports financial support was provided by 10.13039/100016962South Africa Department of Science and Innovation (GB0200065). Marshall Keyster reports financial support was provided by 10.13039/100016962South Africa Department of Science and Innovation (GB0200066). Augustine Innalegwu Daniel reports financial support was provided by Tertiary Education Trust Fund. Marshall Keyster reports financial support was provided by 10.13039/501100001321NRF Centre of Excellence in Food Security (Project ID: 170202). If there are other authors, they declare that they have no known competing financial interests or personal relationships that could have appeared to influence the work reported in this paper.
